# Characterization and Comparative Analysis of Complete Chloroplast Genome of *Vachellia tortilis* subsp. *tortilis* (Fabaceae)

**DOI:** 10.3390/biology15211952

**Published:** 2026-10-30

**Authors:** Mohammad S. Alawfi, Dhafer A. Alzahrani, Enas J. Albokhari, Reem S. Aloufi

**Affiliations:** 1 Department of Biology, College of Sciences, King Khalid University, Abha 61421, Saudi Arabia; 2 Department of Biological Sciences, Faculty of Sciences, King Abdulaziz University, Jeddah 21589, Saudi Arabia; dalzahrani@kau.edu.sa; 3 Department of Biology, College of Science, Umm Al-Qura University, Makkah 24381, Saudi Arabia; ejbokhary@uqu.edu.sa; 4 Independent Researcher, Madinah 42318, Saudi Arabia; reeeem.saud@hotmail.com

**Keywords:** *Vachellia tortilis* subsp. *tortilis*, genus *Vachellia*, Fabaceae family, chloroplast (cp) genome

## Abstract

The *Vachellia tortilis* subsp. *tortilis* is a dryland tree included in the Fabaceae family. The genus *Vachellia* includes 164 species, 38 subspecies, and 25 varieties. The taxonomic placement of *Vachellia* has historically been unstable; the genus was only recently segregated from *Acacia*. In this study, we sequenced and characterized the chloroplast genome of *V. tortilis* subsp. *tortilis* and compared it with those of related species. The total chloroplast genome length of *V. tortilis* subsp. *tortilis* was 164,956 bp, and it contained 132 genes: 87 protein-coding genes, eight ribosomal RNA genes, and 37 transfer RNA genes. Simple repeat analysis showed that the chloroplast genome of *V. tortilis* subsp. *tortilis* contained 273 repeats. Codon usage bias analysis showed that the chloroplast genome of *V. tortilis* subsp. *tortilis* encoded 24,633 codons. The phylogenetic analysis revealed two distinct clades in the genus *Vachellia*: the first comprises *V. tortilis* subsp. *tortilis* and *Vachellia tortilis* subsp. *raddiana*, while the second includes *Vachellia flava*, *Vachellia seyal*, and *Vachellia nilotica*. This study improves knowledge of evolutionary relationships in the genus *Vachellia* and provides valuable genetic resources.

## Simple Summary

The samar tree (*Vachellia tortilis* subsp. *tortilis*) is a tree within the Fabaceae family and is widely distributed in the Middle East and Africa. To better understand the genetic diversity within the genus *Vachellia*, we mapped the complete chloroplast DNA sequence of the samar tree. Chloroplasts are tiny parts of plant cells that turn sunlight into food. The total number of genes within the samar tree is 132, consisting of about 164,956 genetic letters. By comparing this newly mapped chloroplast DNA with that of other *Vachellia* species, we were able to build a detailed phylogenetic tree. This type of data can help us figure out how different species within the genus *Vachellia* are related to one another and how they evolved over time. Ultimately, decoding this genetic information gives scientists valuable tools for future research, including biodiversity conservation strategies, agricultural crop improvement, the discovery of new plant-based medicines, and simply understanding the natural history of these plants.

## 1. Introduction

*Vachellia tortilis*, previously referred to as *Acacia tortilis*, is a dryland tree in the Fabaceae family. The species is recognized as a keystone species in the dryland ecosystems of the Middle East and Africa [1]. It used to be included in the genus *Acacia*, but it is now classified under the genus *Vachellia* in the Mimosoideae subfamily in the Fabaceae family [2]. The genus *Vachellia* was found to be nested in the Mimoseae tribe (in the *Parkia* clade) and includes 164 species, 38 subspecies, and 25 varieties [3,4,5]. Moreover, *V. tortilis* is divided into five subspecies: *tortilis*, *heteracantha*, *spirocarpa*, *raddiana*, and *campoptila* [5].

Morphologically, *Vachellia tortilis* subsp. *tortilis* is a tree (commonly known as the samar tree) that grows from 3 m to 7 m. The leaves are dark green, alternate, and bipinnate with 4–6 pairs, each having 6–10 pairs of leaflets per pinna. The thorns bear pairs of short hooked spines alongside long, straight, and whitish spines. The flowers are highly aromatic and white to yellowish-white and appear round in tight clusters. Usually hairless, the pods are spirally contorted, 5–15 cm long and green to yellowish-brown.

Biodiversity in dryland ecosystems continues to undergo unprecedented degradation and destruction due to the overexploitation of its vegetation [6]. In the face of these threats to biodiversity, *V. tortilis* is one of the few wild *Acacia* that survives in arid lands, which makes it an essential species for conservation [7]. As *V. tortilis* is recognized as one of the most keystone species in dryland ecosystems, there is a need to understand genetic structure at the species level and genetic variation at the subspecies level to assist its conservation.

Genome sequences are relatively stable and offer a data reference and a strong theoretical basis for solving classification problems [8]. In angiosperms, chloroplast (cp) genomes are often maternally inherited, displaying exceptional conservation with little to no intraspecific variation, recombination events, and a relatively small genome size, thus making them an effective method for species identification, phylogenetic analyses, and comparative analysis [9,10,11]. In recent years, cp genome data have played a prominent role in the resolution of complex phylogenetic relationships at various taxonomical levels and can be used as a plant barcode, overcoming traditional DNA barcodes (*rbcL*, *matK*, *ITS*) [12,13]. Moreover, a significant gap exists in the rate of evolutionary progression between gene-coding and non-coding regions in cp genomes, which is ideal for investigating several taxonomic levels, such as the intraspecific level [14,15,16].

The evolutionary relationships of the genus *Vachellia* have attracted much attention, but only a limited number of studies have been conducted using the cp genome [17,18]. More characterization and comparative analysis studies are still needed to clarify the relationships between species and subspecies in this genus. Therefore, we initially performed the sequencing, assembly, and annotation of the cp genome of *V. tortilis* subsp. *tortilis*, followed by the construction of a phylogenetic tree. A comparative analysis was also performed in this study. This comprehensive approach aimed to acquire a deeper understanding of the cp genome features of *V. tortilis* subsp. *tortilis* at the intraspecific level and its related species in the genus *Vachellia*.

## 2. Materials and Methods

### 2.1. Plant Material, DNA Extraction, and Sequencing

The plant material used in this study was fresh leaves collected from the Jabal Uhud/Medina region (24°30′48.0″ N 39°37′04.6″ E) on 18 April 2024 and preserved using silica gel. Only single individual was collected from mid-altitude and semi-arid habitats of Jabal Uhud, identified and verified using the morphological approaches (leaf, stem and seed characteristics) by Dr. Mohammad Alawfi, Department of Biology, King Khalid University, Abha, Saudi Arabia. A voucher specimen (Number: KAU-V24087) has been deposited at the King Abdulaziz University herbarium. DNA extraction, library construction, and genome sequencing were conducted by BGI Genomics Company (Hong Kong) using the following methods: Total DNA was extracted from 20 mg of silica-dried leaf material using A DNeasy Plant Mini Kit (QIAGEN, Hilden, Germany), DNA quality and concentration were evaluated by Qubit fluorometry and agarose gel electrophoresis, and the DNBseq platform was used for library construction and sequencing. A total number of 43,132,243 clean reads (>Q20) were obtained, and the raw dataset was filtered with SOAPnuke software v2.1.9 [19] to eliminate low-quality reads and adaptor sequences, resulting in 28 GB of high-quality, 150 bp paired-end reads.

### 2.2. Assembly and Annotation

Assembly was conducted using NOVOPlasty v4.3.3 [20] with kmer (K-mer = 33). The cp genome of *V. tortilis* subsp. *raddiana* (KY100265) was used as the reference. After assembly, its quality was assessed using QUAST v5.3.0 [21] and sequencing depth was calculated using Unipro UGENE v53.1 [22]. The sequence was annotated using the GeSeq tool [23]. The annotated genome was created using OGDRAW v1.3.1 [24] and deposited in the GenBank database under accession numbers: (PZ387862).

### 2.3. Repeat Sequence, Codon Preference and IR Boundary Regions Analyses

The simple sequence repeats (SSRs) were recognized using MISA software v2.1 [25], and the minimum thresholds were set at 8, 5, 4, 3, 3, and 3 for mon, di, tri, tetra, penta, and hexa, respectively. MEGA v.12.1 [26] was used to analyze the codon usage across the protein-coding sequences (CDSs) and excluding duplicated coding genes and stop codons. The IRscope online program was used to compare and visualize in detail the boundary regions of five *Vachellia* cp genomes [27].

### 2.4. Nucleotide Diversity (π) and Genome Sequence Divergence Analyses

Nucleotide diversity was analyzed using DNAsp v6.12.03 [28], with a step size of 100 bp and a window length of 600 bp. Five *Vachellia* cp genomes were compared using the online program mVISTA v2.0 [29], with the Shuffle-LAGAN model, using the *Vachellia flava* cp genome as a reference.

### 2.5. Phylogenetic Analysis

To provide insight into the phylogeny in the members of the genus *Vachellia*, we constructed phylogenetic trees using the complete cp genome sequences of five *Vachellia* species, namely *V. tortilis* subsp. *tortilis* (PZ387862), *V. tortilis* subsp. *raddiana* (KY100265), *V. flava* (NC_036734), *Vachellia nilotica* (NC_045514) and *Vachellia seyal* (NC_036735), and four outgroup taxa, namely *Acacia crassicarpa* (OK_480071), *Mimosa bimucronata* (OM_687265), *Parkia timoriana* (OK662459) and *Senegalia senegal* (MK645903) (Table 1). The sequences were aligned using MAFFT v.7.313 [30], and the phylogenetic tree was inferred using the maximum likelihood (ML) method. ML trees were constructed using IQ-TREE v.2.2.2.6 [31], with the TVM + F + I + G4 model and 10,000 ultrafast bootstrap replications.

## 3. Results

### 3.1. Characteristics of the V. tortilis subsp. tortilis Chloroplast Genome

**Figure 1:**
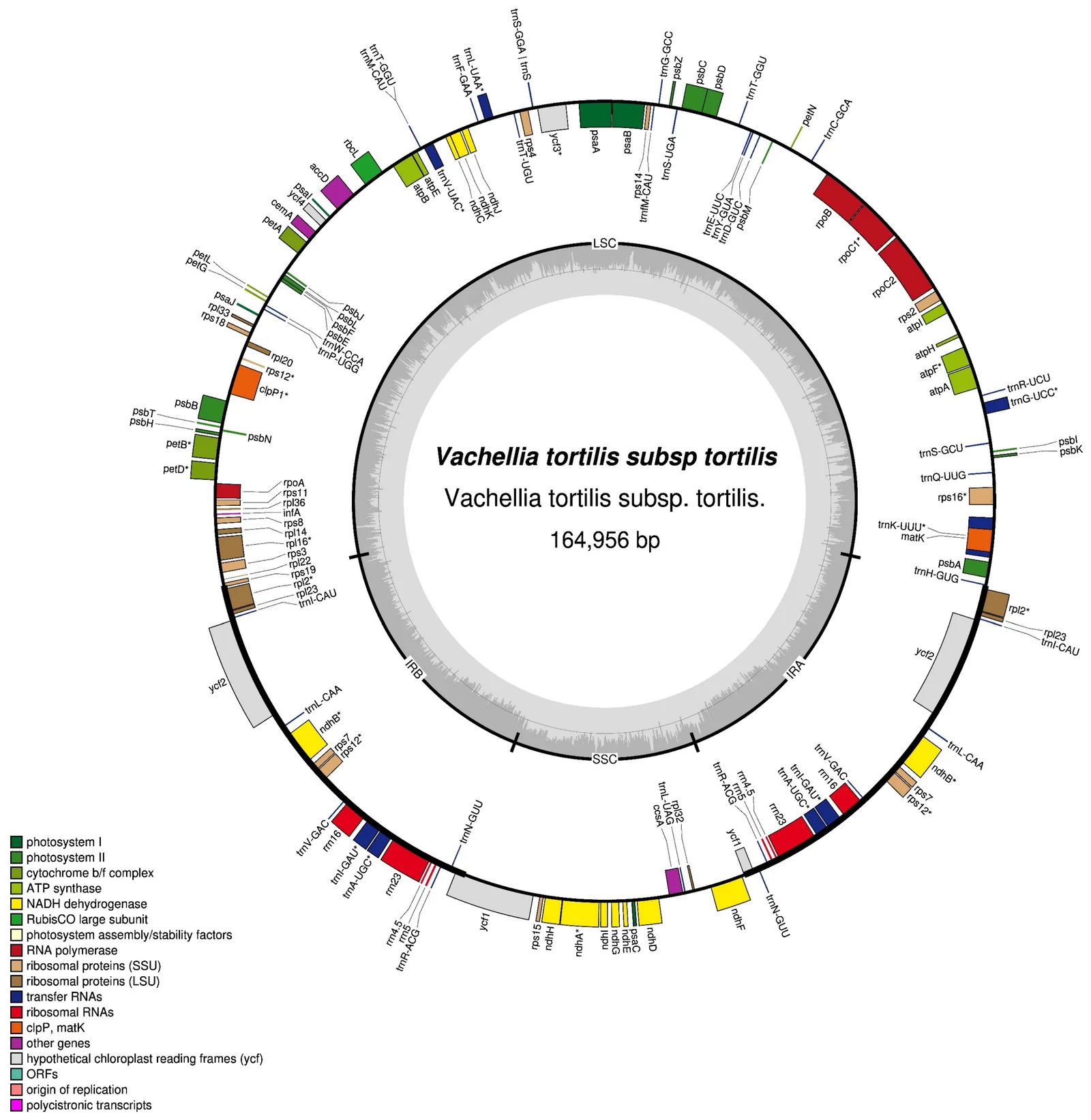
The cp genome map of *V. tortilis* subsp. *tortilis*. The transcription of the genes located inside the circles is clockwise. Genes marked with an asterisk contain introns. The transcription of genes located outside of the circles occurs counterclockwise. Functional genes are shown by the colored bars.

The cp genome of *V. tortilis* subsp. *tortilis* was 164,956 bp in size (Figure 1), with circular and quadripartite structures comprising a pair of inverted repeat (IR) regions (26,049 bp) each, a large single-copy (LSC) region (93,658 bp), and a small single-copy (SSC) region (19,200 bp). The total GC content in the *V. tortilis* subsp. *tortilis* cp genome was 35.38%. The GC contents of IR, LSC, and SSC were 42.67%, 32.5%, and 29.65%, respectively (Table 2). The average sequencing depth was 9798×, with a maximum depth of 26,356× and a minimum depth of 285×. The number of genes in the *V. tortilis* subsp. *tortilis* cp genome was 132 in total (113 unique genes), comprising 87 protein-coding genes, eight ribosomal RNA genes and 37 transfer RNA genes. Eighteen genes were identified as duplicated in two IR regions, comprising seven protein-coding genes *(ndhB*, *rpl2*, *rpl23*, *rps7*, *rps12*, *ycf1*, and *ycf2*), four rRNA genes (*rrn4.5*, *rrn5*, *rrn16*, and *rrn23*), and seven tRNA genes (*trnA-UGC*, *trnI-CAU*, *trnI-GAU*, *trnL-CAA*, *trnN-GUU*, *trnR-ACG*, and *trnV-GAC*). The *rps12* gene appears in IRa and IRb regions, with its third copy found in the LSC region. The genes can be classified into four distinct categories based on function: self-replication genes, photosynthesis genes, other genes, and genes of unknown function (Table S1). The cp genome of *V. tortilis* subsp. *tortilis* comprises 18 introns in several CDSs and tRNA genes (Table S2). The *ycf3* and *clpP1* genes comprise more than one intron, while the *trnK-UUU* gene contains the largest intron, with a length of 2587 bp in size.

### 3.2. Simple Sequence Repeats, Codon Preference and IR Boundary Regions

SSRs, which are polymorphous and reliable, are considered typical molecular markers for species identification and phylogenetic analyses. In this study, the number of SSRs in the *V. tortilis* subsp. *tortilis* cp genome was 273 repeats and was mainly observed in the LSC region. These SSRs consisted of 235 mononucleotide repeats, 11 dinucleotide repeats, 14 trinucleotide repeats, 11 tetranucleotide repeats, one pentanucleotide repeat, and one hexanucleotide repeat (Table 3). These repeat units consisted mostly of adenine (A) and thymine (T), with mononucleotide A/T repeats representing the most frequent repeats, followed by trinucleotide AAT/ATT repeats (Table 3). Moreover, SSRs were distributed as follows: 216 SSRs (79.1%) at the LSC region, 47 SSRs (17.2%) at the SSC region and 10 SSRs (3.7%) at IR regions. The longest SSR was 18 bp in size and found in the LSC region, while the shortest SSR was 8 bp in size and distributed across the LSC and SSC regions.

**Figure 2:**
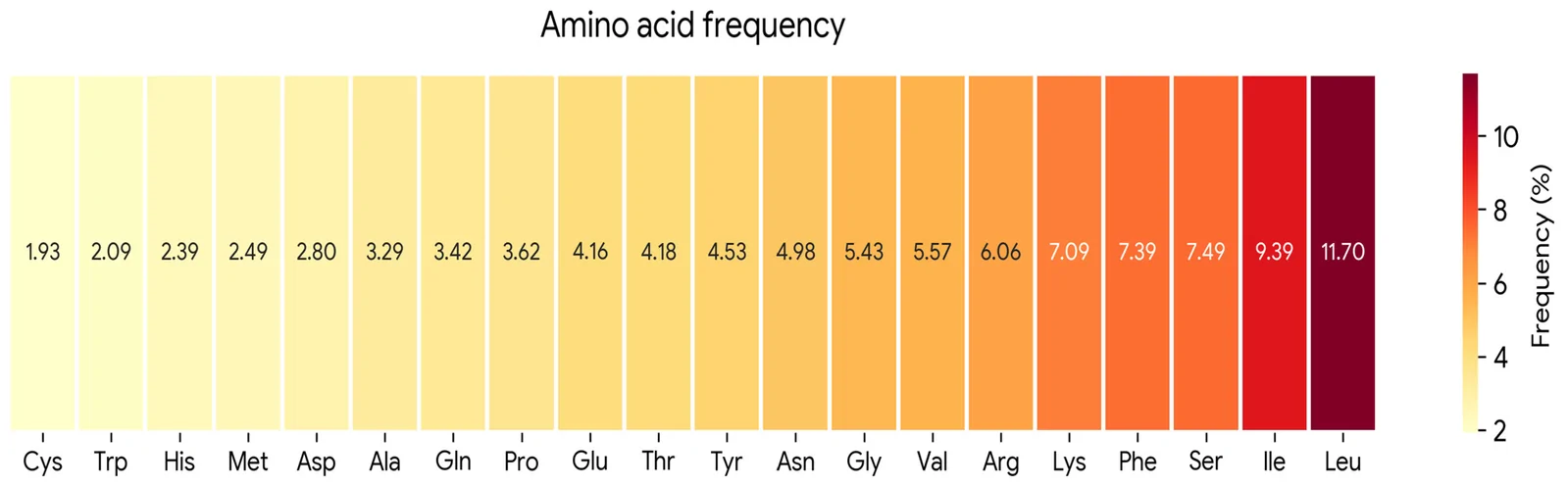
Amino acid frequency heat map in the cp genome of *V. tortilis* subsp. *tortilis*.

Codon usage bias (CUB) refers to the phenomenon that strongly affects the accuracy and efficiency of protein synthesis. In this study, the CUB of CDSs was examined, and the analysis showed that the *V. tortilis* subsp. *tortilis* cp genome encoded 24,633 codons. In terms of amino acid content, leucine was the most abundant codon (11.70%), while cysteine was the least abundant amino acid (1.93%) (Figure 2). The relatively synonymous codon usage (RSCU) examination showed that 28 codons had an RSCU value greater than 1 and that 92.8% of these codons ended with an A or U nucleotide base. By contrast, 31 codons were observed to have an RSCU value less than 1, and 83.8% of these codons ended with a C or G nucleotide base. Only three codons were neutral, with an RSCU value equal to 1, and all of these codons ended with a C or G nucleotide base. The AGA (Arg) is the only codon that is overrepresented, with an RSCU value greater than 2 (Table S3).

**Figure 3:**
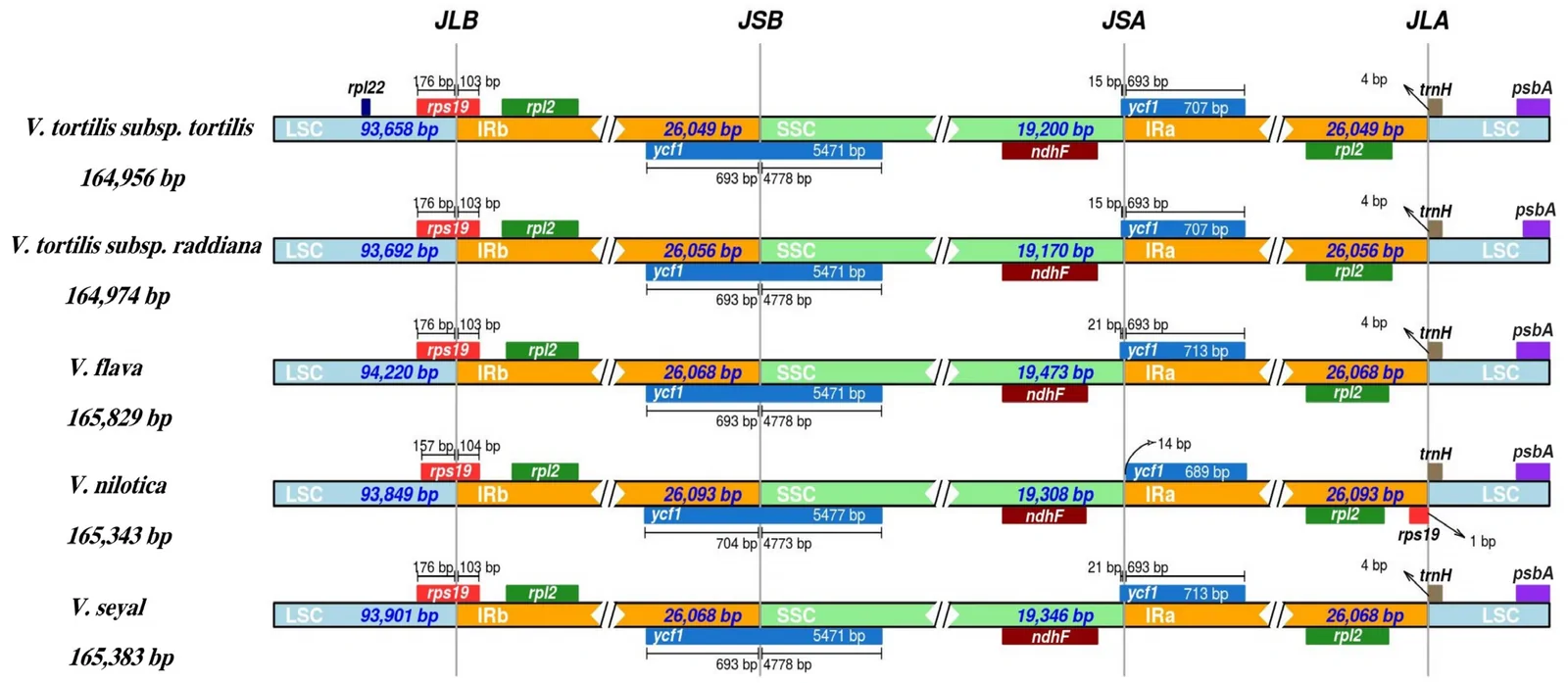
Comparison of the borders of the inverted repeat (IR) regions, small single-copy (SSC) region and large single-copy (LSC) region between the five cp genomes of *Vachellia* species.

The boundaries of the IR, LSC, and SSC regions were compared between the assembled cp genome of *V. tortilis* subsp. *tortilis* (PZ387862) and the following cp genomes taxa: *V. tortilis* subsp. *raddiana* (KY100265), *V. flava* (NC_036734), *V. nilotica* (NC_045514), and *V. seyal* (NC_036735). The lengths of the IR, LSC, and SSC regions were similar among the five genomes (Figure 3), reflecting structural stability. However, a few structural variations were observed: the *rpl22* gene was found in the LSC region in *V. tortilis* subsp. *tortilis* but was absent in the other taxa, the IRa/LSC junction in *V. nilotica* involved the *rps19* gene, and the *ycf1* gene was found only in the IRa region in *V. nilotica*, while it was located at the boundary of the IRa/SSC junction in the other taxa.

### 3.3. Nucleotide Diversity (π) and Genome Sequence Divergence

**Figure 4:**
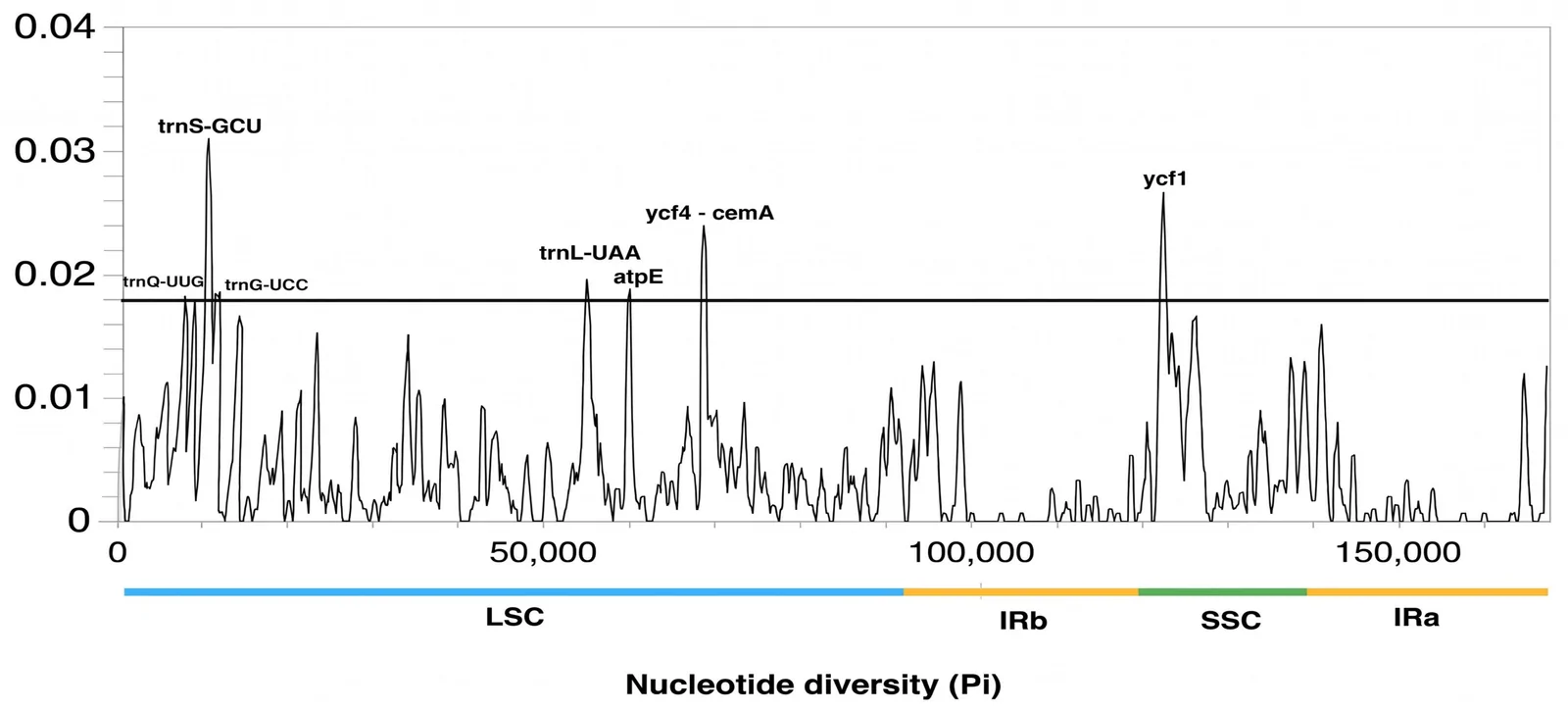
Nucleotide diversity values among cp genomes of *V. tortilis* subsp. *tortilis*, *V. tortilis* subsp. *raddiana*, *V. flava*, *V. seyal* and *V. nilotica*. Variation hotspots (Pi > 0.018) are labeled above the corresponding gene position.

The identification of nucleotide diversity (π) provides a basis for the design of specific primers or barcodes that enhance resolution in species delimitation and phylogenetic studies. Nucleotide diversity analysis was conducted using five species within *Vachellia*. The π values ranged from 0.00000 to 0.03100 (Figure 4). A total of eight highly variable regions (π ≥ 0.018) were identified: *trnQ-UUG* (0.01833), *trnS-GCU* (0.03100), *trnG-UCC* (0.01867), *trnL-UAA* (0.01967), *atpE* (0.01900), *ycf4* (0.02400), and *cemA* (0.02233) in the LSC region, and *ycf1* (0.02667) in the SSC region.

**Figure 5:**
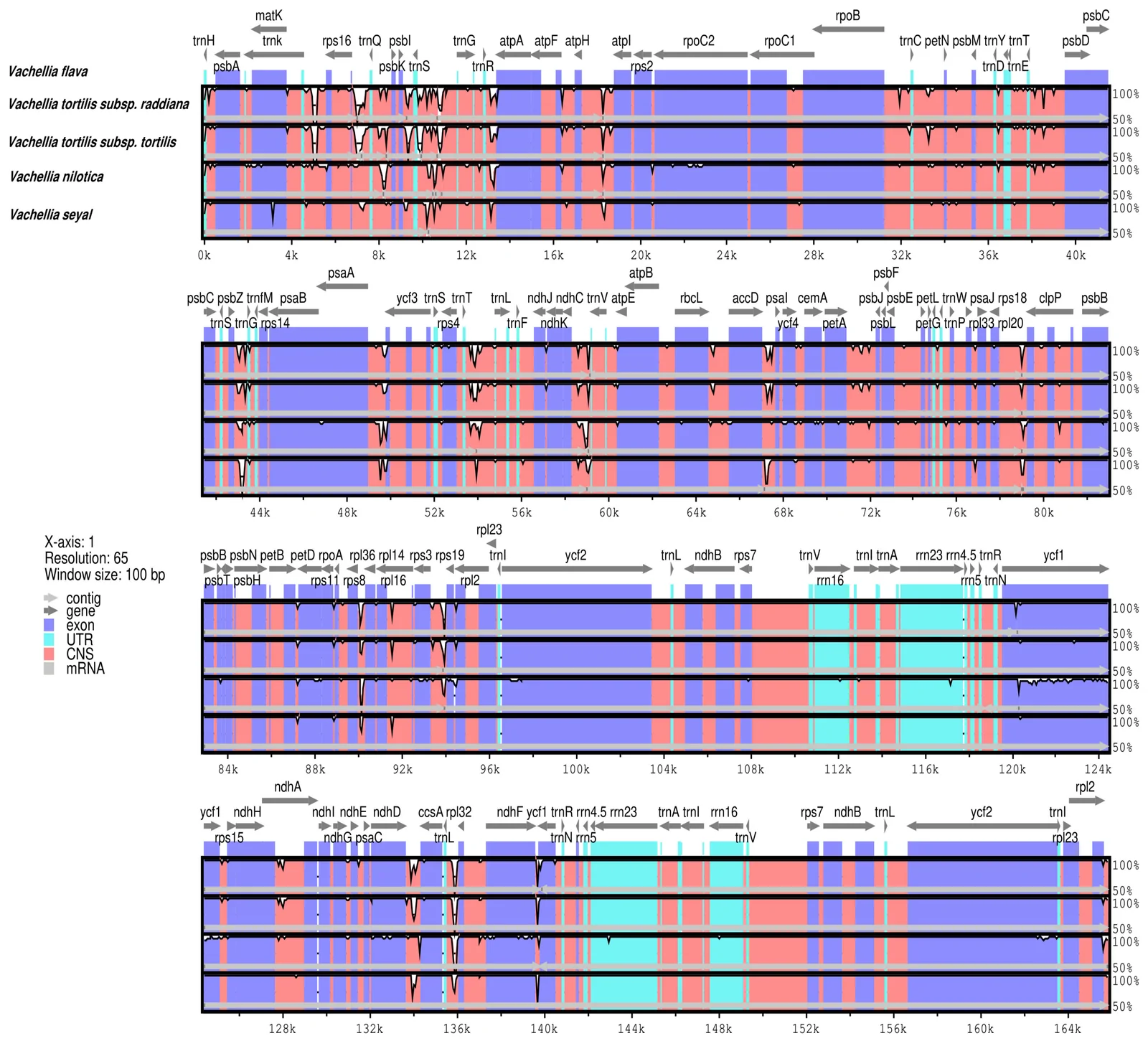
Five *Vachellia* taxa cp genomes were visually aligned using *V. flava* as a reference. The x-axis displays the cp genome coordinate, and the y-axis shows the identity percentage, which ranges from 50% to 100%. The upper arrows show the direction of each gene. UTR stands for untranslated regions, and CNS for conserved non-coding regions. The sequence alignment was performed using the mVISTA software.

A comparative analysis was performed to identify sequence divergence regions among five *Vachellia* taxa, using *Vachellia flava* as the reference genome (Figure 5). The analysis revealed that noncoding regions exhibited greater nucleotide divergence than coding regions. The genes that showed nucleotide divergence within the coding regions included *matK*, *trnK*, *rpoC2*, *psaA*, *ycf1*, *ycf2*, *ndhD*, *ccsA*, and *ndhF*. *V. nilotica* had the greatest sequence divergence among the *Vachellia* taxa, whereas *V. tortilis* subsp. *tortilis* and *V. tortilis* subsp. *raddiana* were almost identical in terms of sequence divergence.

### 3.4. Phylogenetic Tree

**Figure 6:**
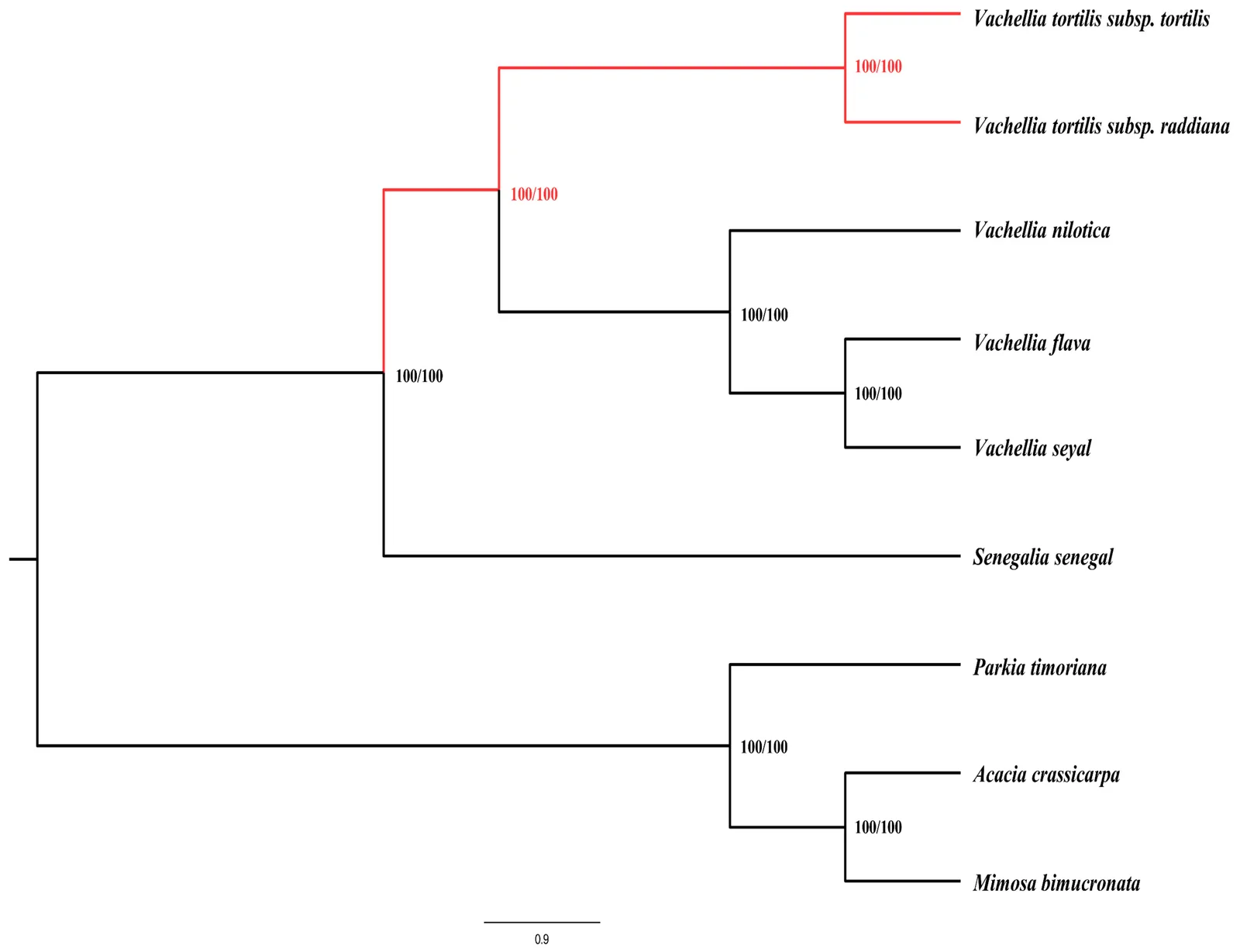
Phylogenetic tree showing the relationships among five *Vachellia* taxa along with four other Fabaceae taxa, produced by ML analysis of 9 cp genomes. The branch node numbers represent the (BS) values.

To clarify evolutionary relationships among *Vachellia* taxa, a phylogenetic analysis was reconstructed using the cp genome sequences of five *Vachellia* taxa along with four other Fabaceae taxa. The results showed that the *Vachellia* taxa were classified into two clades. One clade comprised *V. tortilis* subsp. *tortilis* and *V. tortilis* subsp. *raddiana*. The second clade was divided into two subclades: the first included *V. flava* and *V. seyal*, while the second contained only *V. nilotica*. *Senegalia senegal* was positioned as the sister clade to the *Vachellia* clade, while *Acacia crassicarpa*, *Mimosa bimucronata*, and *Parkia timoriana* demonstrated the most distant relationships (Figure 6). All branches showed full bootstrap support.

## 4. Discussion

This study reports the complete cp genome of *V. tortilis* subsp. *tortilis*. The cp genome was 164,956 bp in length, encoded 132 genes, and had a GC content of 35.38%. Moreover, it displayed the typical conserved quadripartite structure observed in other angiosperm genomes [32,33]. The highest GC content was observed in the IR regions (42.67%), which may be due to their structural stability and the presence of all ribosomal RNA (rRNAs) genes within these regions [34,35]. A total of 18 of the 113 genes contained introns. Intron content is highly conserved within angiosperm cp genomes, with the majority of genomes containing 18 intron-bearing genes [36], which play an important role in regulating gene expression [37]. The SSR analysis revealed that the cp genome of *V. tortilis* subsp. *tortilis* included 273 repeats. SSRs have been used extensively as molecular markers in taxonomic studies [38]. Mononucleotide repeats were the dominant type, contributing to AT richness. These findings are consistent with those reported for most angiosperm cp genomes [39,40]. Because of their high mutation rates, SSRs represent valuable molecular markers for assessing genetic diversity and supporting biodiversity conservation efforts [41].

The CUB analysis indicated that the genome encoded 24,633 codons. CUB reflects the origins of genes or species, their temporal changes and evolutionary processes, highlighting genetic variations among organisms [42]. The RSCU analysis identified 28 codons with RSCU > 1, 31 codons with RSCU < 1 and only three codons with RSCU = 1. The cp genome exhibits high codon usage bias [43], driven by a balance between natural selection and mutational bias [44], leading to a preference for A/T-ending codons due to low GC content. Expansion and contraction at the boundaries of inverted repeat regions are common in angiosperm cp genomes and may result in genome size variation, gene duplication or deletion, and the formation of pseudogenes [45]. The IR regions of *V. tortilis* subsp. *tortilis* and four *Vachellia* taxa were compared; all five cp genomes displayed significant conservation. However, the *rpl22* gene was detected in the LSC region of the cp genome of *V. tortilis* subsp. *tortilis* and absent in the other *Vachellia* taxa. The *rpl22* gene has been reported as a pseudogene gene in some taxa in Fabaceae [46].

Nucleotide diversity analysis of the five *Vachellia* cp genomes indicated that π values ranged from 0.00000 to 0.03100. Moreover, eight mutated hotspots were identified. Among intergenic regions, the *trnS-GCU* interval exhibited the highest nucleotide diversity (π = 0.03100), while among coding regions, the *ycf1* interval displayed the highest nucleotide diversity (π = 0.02667). These regions are likely to show a rise in nucleotide substitutions and may serve as effective DNA barcodes for species-level identification. For example, the *ycf1* gene has been considered one of the most variable regions of the cp genome and is widely used as a DNA barcode in land plants [47]. Sequence divergence analysis indicated that all five *Vachellia* cp genomes were highly conserved; however, a few variable regions were detected in *matK*, *trnK*, *rpoC2*, *psaA*, *ycf1*, *ycf2*, *ndhD*, *ccsA*, and *ndhF* genes. Some of these markers have been used in different phylogenetic studies of flowering plants [48,49,50]. The use of these high-diversity regions as species-specific DNA barcodes within the genus *Vachellia* may be valuable.

The evolutionary relationship within the genus *Vachellia* (Fabaceae) has not been fully resolved previously, relying solely on a limited number of chloroplast markers [51,52]. Cp genomes are widely used in phylogenomic studies to explore relatedness, classify species [53], and resolve complex relationships at the species and subspecies levels, as demonstrated in *Eriobotrya* species [54]. Phylogenetic analysis based on cp genomes showed full congruence among the five *Vachellia* taxa and classified them into two clades. The first included the two subspecies *V. tortilis* subsp. *tortilis* and *V. tortilis* subsp. *raddiana*, while the second included *V. flava*, *V. seyal* and *V. nilotica.* These findings are consistent with the results of prior studies [17,18]. The clade containing *Senegalia senegal* was sister to the *Vachellia* clade, also consistent with the results of a previous study [18]. These results support the current placement of taxa within the genus *Vachellia* and reject the previous classification of these species within the genus *Acacia*.

## 5. Conclusions

In this study, the basic characteristics of *V. tortilis* subsp. *tortilis* were characterized and compared. Moreover, base composition, SSRs, codon usage, IR boundaries, nucleotide diversity (π), genome sequence divergence, and phylogenetic relationships were analyzed and compared. The cp genome of *V. tortilis* subsp. *tortilis* was 164,956 bp in length, with 132 genes in total, including 273 SSRs, which were mostly mononucleotides (A/T repeats). Moreover, the genome encoded 24,633 codons, the π values ranged from 0.00000 to 0.03100, and genes that showed nucleotide divergence, including *matK*, *trnK*, *rpoC2*, *psaA*, *ycf1*, *ycf2*, *ndhD*, *ccsA*, and *ndhF*, were identified as the most variable regions. A few variations in structure have been observed: In *V. tortilis subsp. tortilis*, the *rpl22* gene was found in the LSC region; however, it was completely absent in the other *Vachellia* taxa. In the phylogenetic tree, the genus *Vachellia* was classified into two clades: the first represented by *V. tortilis* subsp. *tortilis* and *V. tortilis* subsp. *raddiana*, and the second represented by *V. flava*, *V. seyal*, and *V. nilotica*. *Senegalia senegal* was recovered as the sister clade to the *Vachellia* clade. The findings of this study validate the current classification of these taxa within the genus *Vachellia* rather than within the genus *Acacia*. Further cp genome sequencing of *Vachellia* species is recommended to strengthen comparative analyses, develop species-specific markers, and improve the understanding of evolutionary relationships within the genus.

## Figures and Tables

**Table 1:** GenBank accession numbers and taxonomic affiliation of the cp genomes used in the phylogenetic analysis of the genus *Vachellia*.

	Family	Species	Accession Number
1	Fabaceae	*Vachellia tortilis* subsp. *tortilis*	PZ387862
2	Fabaceae	*Vachellia tortilis* subsp. *raddiana*	KY100265
3	Fabaceae	*Vachellia flava*	NC_036734
4	Fabaceae	*Vachellia nilotica*	NC_045514
5	Fabaceae	*Vachellia seyal*	NC_036735
6	Fabaceae	*Acacia crassicarpa*	OK_480071
7	Fabaceae	*Mimosa bimucronata*	OM_687265
8	Fabaceae	*Parkia timoriana*	OK662459
9	Fabaceae	*Senegalia senegal*	MK645903

**Table 2:** Base composition and genome structural features of the cp genome of *V. tortilis* subsp. *tortilis*.

Genome size (bp)	164,956
Inverted repeat (IR) regions bp	26,049
Large single-copy (LSC) region bp	93,658
Small single-copy (SSC) region bp	19,200
Total number of genes	132
rRNA	8
tRNA	37
Protein-coding genes	87
T (U) %	32.77
C %	17.88
A %	31.83
G %	17.50
Overall GC content %	35.38
GC content in LSC %	32.5
GC content in SSC %	29.65
GC content in IR %	42.67

**Table 3:** Simple sequence repeats (SSRs) identified in the chloroplast genome of *V. tortilis* subsp. *tortilis*.

SSR Type	Repeat Unit	Total
Mono	A/T	231
	C/G	4
Di	AG/CT	2
	AT/AT	9
Tri	AAT/ATT	12
	ACT/AGT	1
	ATC/ATG	1
Tetra	AAAC/GTTT	1
	AAAT/ATTT	5
	AATC/ATTG	1
	AATG/ATTC	1
	AATT/AATT	3
Penta	AAAAT/ATTTT	1
Hexa	AATTAT/AATTAT	1

## Data Availability

The datasets generated and analyzed in this study are available in the GenBank of NCBI, and the complete chloroplast sequence of *V. tortilis* subsp. *tortilis* deposited in GenBank of NCBI under the following accession number: (PZ387862).

## Supplementary Materials

The following supporting information can be downloaded at: https://www.mdpi.com/article/10.3390/biology15211952/s1, Table S1: Genes contents in *Vachellia tortilis* subsp. *tortilis* chloroplast genome; Table S2: Length of introns and exons in *Vachellia tortilis* subsp. *tortilis* chloroplast genome; Table S3: Codon-anticodon recognition patterns and codon usage of the *Vachellia tortilis* subsp. *tortilis* chloroplast genome.
