# Supplementary material for: Characterization and Comparative Analysis of Complete Chloroplast Genome of Vachellia tortilis subsp. tortilis (Fabaceae)
Source: Biology (Basel). 2026 Oct 30;15(21):1952. doi: 10.3390/biology15211952 (PMC13761856; doi:10.3390/biology15211952)
Supplement: Supplementary file 1 [file biology-15-01952-s001.zip › biology-4633687-supplementary.pdf]

Supplementary

Table S1. Genes contents in *Vachellia tortilis* subsp. *tortilis* chloroplast genome.

Category	Gene groups	Gene Names
**RNA genes**	Transfer RNA genes (tRNA)	*trnH-GUG, trnK-UUU +, trnQ-UUG, trnS-GCU, trnG-UCC +, trnR-UCU, trnC-GCA, trnD-GUC, trnY-GUA, trnE-UUC, trnT-GGU, trnS-UGA, trnG-GCC, trnfM-CAU, trnS-GGA, trnL-UAA +,trnF-GAA, trnV-UAC +, trnT-CAU, trnM-CAU, trnW-CCA, trnP-UGG, trnI-CAU a, trnL-CAA a, trnV-GAC a, trnI-GAU +a, trnA-UGC +a, trnR-ACG a, trnN-GUU a, trnL-UAG.*
	Ribosomal RNA genes (rRNA)	*rrn4.5 a, rrn5 a, rrn16 a, rrn23 a*
**Ribosomal proteins**	Small sub-unit of ribosome	*rps2, rps3, rps4, rps7a, rps8, rps11, rps12+a, rps14, rps15, rps16+, rps18, rps19*
**Transcription Genes**	Large sub-unit of ribosome	*rpl2+a, rpl14, rpl16+, rpl20, rpl22, rpl23a, rpl32, rpl33, rpl36*
	DNA-dependent RNA polymerase	*rpoA, rpoB, rpoC1+, rpoC2*
**Protein-coding genes**	Photosystem I	*psaA, psaB, psaC, psaI, psaJ, ycf3++*
	Photosystem II	*psbA, psbB, psbC, psbD, psbE, psbF, psbH, psbI, psbJ, psbK, psbL, psbM, psbN, psbT, psbZ*
	Sub-unit of cytochrome	*petA, petB+, petD+, petG, petL, petN*
	Sub-unit of synthase	*atpA, atpB, atpE, atpF+, atpH, atpI*
	Large sub-unit of rubisco	*rbcL*
	NADH dehydrogenase	*ndhA+, ndhB+a, ndhC, ndhD, ndhE, ndhF, ndhG, ndhH, ndhI, ndhJ, ndhK*
	ATP-dependent protease subunit P	*clpP++*
**Other genes**	Cp envelope membrane protein	*cemA*
	C-type cytochrome systhesis	*ccsA*
	Translation initiation factor,	*infA*
	Maturase	*matK*
	Sub-unit acetyl-coA carboxylase	*accD*
	Hypothetical proteins	*ycf2a, ycf4*
	Component of TIC complex	*ycf1a*

* *a* Gene with multiple copies, +Gene with one intron and ++Gene with two introns

Table S2. Length of introns and exons in *Vachellia tortilis* subsp. *tortilis* chloroplast genome.

Gene	Location	Exon I (bp)	Intron I (bp)	Exon II (bp)	Intron II (bp)	Exon III (bp)
trnK-UUU	LSC	34	2587	36		
rps16	LSC	244	898	39		
trnG-UCC	LSC	20	706	50		
atpF	LSC	407	729	143		
rpoC1	LSC	1618	824	429		
ycf3	LSC	152	737	229	725	123
trnL-UAA	LSC	34	533	49		
trnV-UAC	LSC	36	618	38		
clpP1	LSC	224	689	293	791	78
petB	LSC	5	800	641		
petD	LSC	7	658	474		
rpl16	LSC	398	1157	8		
rpl2	IR	433	666	390		
ndhB	IR	755	691	776		
rps12	IR	26	535	230		
trnI-GAU	IR	41	949	34		
trnA-UGC	IR	37	803	34		
ndhA	SSC	552	1386	538		

Table S3. Codon-anticodon recognition patterns and codon usage of the *Vachellia tortilis* subsp. *tortilis* chloroplast genome.

Amino Acid	Codon	RSCU	Count	Amino Acid	Codon	RSCU	Count
Phe	UUU(F)<br>UUC(F)	1.2<br>0.8	1090<br>730	Tyr	UAU(Y)<br>UAC(Y)	1.35<br>0.65	752<br>364
Leu	UUA(L)<br>UUG(L)<br>CUU(L)<br>CUC(L)<br>CUA(L)<br>CUG(L)	1.44<br>1.33<br>1.05<br>0.6<br>0.96<br>0.63	694<br>638<br>502<br>286<br>461<br>301	Gly	GGU(G)<br>GGC(G)<br>GGA(G)<br>GGG(G)	1.12<br>0.49<br>1.44<br>0.94	375<br>164<br>483<br>316
Ile	AUU(I)<br>AUC(I)<br>AUA(I)	1.26<br>0.74<br>1.01	969<br>569<br>775	His	CAU(H)<br>CAC(H)	1.38<br>0.62	406<br>183
Met	AUG(M)	1	614	Trp	UGG(W)	1	515
Val	GUU(V)<br>GUC(V)<br>GUA(V)<br>GUG(V)	1.27<br>0.63<br>1.37<br>0.73	435<br>215<br>471<br>250	Asn	AAU(N)<br>AAC(N)	1.34<br>0.66	822<br>404
Ser	UCU(S)<br>UCC(S)<br>UCA(S)<br>UCG(S)	1.42<br>1<br>1.33<br>0.73	438<br>308<br>410<br>224	Lys	AAA(K)<br>AAG(K)	1.32<br>0.68	1154<br>593
Pro	CCU(P)<br>CCC(P)<br>CCA(P)<br>CCG(P)	1.15<br>0.69<br>1.27<br>0.88	257<br>154<br>284<br>197	Asp	GAU(D)<br>GAC(D)	1.47<br>0.53	505<br>184
Thr	ACU(T)<br>ACC(T)<br>ACA(T)<br>ACG(T)	1,21<br>0.86<br>1.34<br>0.59	311<br>222<br>344<br>153	Glu	GAA(E)<br>GAG(E)	1.34<br>0.66	687<br>338
Ala	GCU(A)<br>GCC(A)<br>GCA(A)<br>GCG(A)	1.52<br>0.65<br>1.2<br>0.63	308<br>132<br>244<br>127	Cys	UGU(C)<br>UGC(C)	1.14<br>0.86	271<br>204
Ser	AGU(S)<br>AGC(S)	0.89<br>0.62	275<br>190	Gln	CAA(Q)<br>CAG(Q)	1.27<br>0.73	535<br>308
Arg	AGA(R)<br>AGG(R)	2.06<br>1.09	513<br>271	Arg	CGU(R)<br>CGC(R)<br>CGA(R)<br>CGG(R)	0.69<br>0.4<br>0.98<br>0.78	171<br>99<br>244<br>194
